# Phantom-based Quality Assurance of a Clinical Dose Accumulation Technique Used in an Online Adaptive Radiation Therapy Platform

**DOI:** 10.1016/j.adro.2022.101138

**Published:** 2022-12-06

**Authors:** Borna Maraghechi, Thomas Mazur, Dao Lam, Alex Price, Lauren Henke, Hyun Kim, Geoffrey D. Hugo, Bin Cai

**Affiliations:** a Department of Radiation Oncology, Washington University, St Louis, Missouri; b Department of Radiation Oncology, University of Texas Southwestern Medical Center, Dallas, Texas

## Abstract

**Purpose:**

This study aimed to develop a routine quality assurance method for a dose accumulation technique provided by a radiation therapy platform for online treatment adaptation.

**Methods and Materials:**

Two commonly used phantoms were selected for the dose accumulation QA: Electron density and anthropomorphic pelvis. On a computed tomography (CT) scan of the electron density phantom, 1 target (gross tumor volume [GTV]; insert at 6 o'clock), a subvolume within this target, and 7 organs at risk (OARs; other inserts) were contoured in the treatment planning system (TPS). Two adaptation sessions were performed in which the GTV was recontoured, first at 7 o'clock and then at 5 o'clock. The accumulated dose was exported from the TPS after delivery. Deformable vector fields were also exported to manually accumulate doses for comparison. For the pelvis phantom, synthetic Gaussian deformations were applied to the planning CT image to simulate organ changes. Two single-fraction adaptive plans were created based on the deformed planning CT and cone beam CT images acquired onboard the radiation therapy platform. A manual dose accumulation was performed after delivery using the exported deformable vector fields for comparison with the system-generated result.

**Results:**

All plans were successfully delivered, and the accumulated dose was both manually calculated and derived from the TPS. For the electron density phantom, the average mean dose differences in the GTV, boost volume, and OARs 1 to 7 were 0.0%, –0.2%, 92.0%, 78.4%, 1.8%, 1.9%, 0.0%, 0.0%, and 2.3%, respectively, between the manually summed and platform-accumulated doses. The gamma passing rates for the 3-dimensional dose comparison between the manually generated and TPS-provided dose accumulations were >99% for both phantoms.

**Conclusions:**

This study demonstrated agreement between manually obtained and TPS-generated accumulated doses in terms of both mean structure doses and local 3-dimensional dose distributions. Large disagreements were observed for OAR1 and OAR2 defined on the electron density phantom due to OARs having lower deformation priority over the target in addition to artificially large changes in position induced for these structures fraction-by-fraction. The tests applied in this study to a commercial platform provide a straightforward approach toward the development of routine quality assurance of dose accumulation in online adaptation.

## Introduction

External beam radiation therapy (RT) plans are typically generated based on a snapshot of a patient's anatomy at the time of pretreatment simulation. However, the positions and shapes of the target and organs at risk (OARs) may vary during the course of radiation treatment. Therefore, the delivery accuracy and plan quality might be compromised because of these interfractional variations. Recently, online adaptive RT (ART), in which a new plan is generated based on a patient's daily anatomy, has shown promising results and is considered a solution to compensate for anatomic differences between an initial scan and a scan acquired immediately before treatment. Plan adaptations also enable the possibility for margin reduction and dose escalation, which could further improve the therapeutic ratio for several disease sites.^1^, ^2^, ^3^, ^4^

Although promising results have been produced, online ART has several limitations. Often, online ART is employed without considering the previously treated fractional dose to the target and OARs. When accounting for previously delivered, accumulated doses may enable more flexibility in adapting a plan. However, the accurate determination of this accumulated dose to both target and OARs, accounting for day-to-day changes in both patient anatomy and delivered plan, is a significant challenge. Because of the complicated shape changes of targets and OARs, deformable image registration (DIR) across treatment fractions is often required to determine the total dose delivered to these structures. Therefore, the accuracy of dose accumulation depends on the integrity of the deformable vector field (DVF) provided by the DIR algorithm in the RT platform.

DIR has been studied extensively in the context of RT, with a multitude of algorithms proposed and used for various applications.^5^, ^6^, ^7^, ^8^, ^9^ Additionally, many studies have investigated the appropriate metrics to evaluate applications of these algorithms.^9^, ^10^, ^11^, ^12^, ^13^, ^14^, ^15^ Certain metrics directly compare reference and warped volumes,^16^, ^17^, ^18^ while others measure registration accuracy for landmarks or contours.^13^^,^^19^, ^20^, ^21^, ^22^, ^23^ Although useful for image-based DIR applications (eg, auto-segmentation), these metrics do not directly provide insight on the fidelity of an accumulated dose volume. Most efforts to quantifiably assess DIR in application to dose accumulation have relied on custom deformable phantoms with integrated, yet complicated, dosimeter arrangements.^24^, ^25^, ^26^, ^27^ Although valuable to mimic realistic motions and probe algorithm limitations, such phantoms may not be suitable for the widespread and routine quality assurance (QA) of commercial packages. In this study, we instead simulate online adaptation with dose accumulation using a common electron density phantom with removable inserts. By simply shuffling insert positions, structure-by-structure correspondence can be established to evaluate consistency of both a DVF and associated accumulated dose volume. A straightforward approach such as this could serve as a check to verify consistency of performance at periodic intervals and after major upgrades or service.

We applied this approach to a recently implemented online adaptive RT platform (Ethos, Varian Medical Systems) capable of plan adaptation based on cone beam computed tomography (CBCT) imaging. The platform leverages structure-guided deformation between CBCT images and the planning CT (pCT) for dose accumulation. In this study, we aim to introduce the dose accumulation algorithm used in Ethos, and present simple periodic QA tests for system consistency checks.

## Methods and Materials

### Ethos dose accumulation

**Figure 1:**
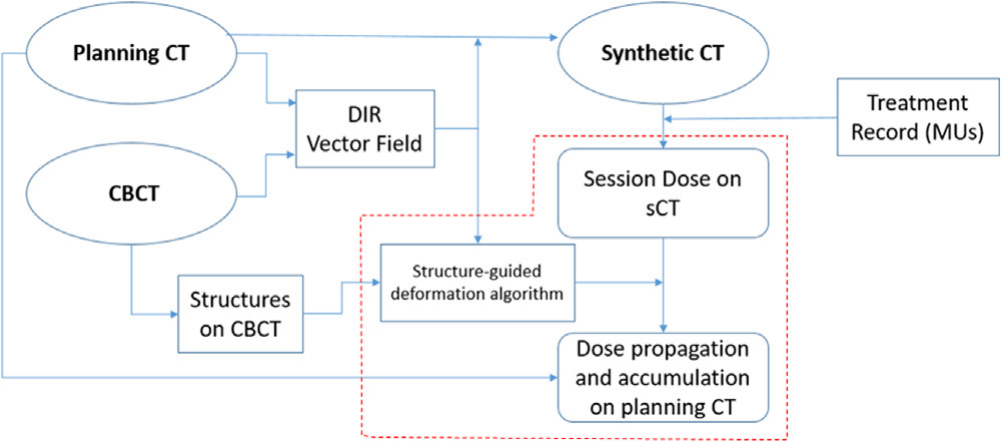
Ethos workflow of session dose generation and dose accumulation on planning computed tomography.

Figure 1 illustrates how session dose is calculated and propagated back to the pCT for dose accumulation, as done in the Ethos system. The system registers the pCT to the fraction CBCT by deriving a rigid transformation for initial alignment, and then applying DIR to produce a DVF.^28^ Using the derived registration matrix and field, the system generates a synthetic CT (sCT) from the pCT that mimics the CBCT for robust dose calculation. For each delivered fraction, the Ethos system recomputes the adapted plan on the sCT to produce the reconstructed or session dose. Simultaneously during the adaptive session, contours are initially mapped to the CBCT based on the DIR, and then manually updated by the physician. The system later performs structure-guided dose deformation to propagate the session dose back to the pCT to produce the propagated dose. Then, the propagated doses are summed over all delivered fractions to obtain the accumulated dose.

### Quality assurance tests

Measurements were carried out using cylindrical and anthropomorphic phantoms. The first set of measurements were performed using a commercial electron density phantom with cylindrical and removable modules or inserts that were defined as target and OARs. Online adaptation in this case was triggered by intentionally contouring the target or OARs at different locations. As the phantom remains rigid in this case, these measurements were primarily aimed at assessing structure guidance in the DIR underlying the dose accumulation. With the anthropomorphic phantom, dose accumulation was tested by synthetically introducing anatomic deformation on the pCT for the sake of simulating a more realistic clinical scenario.

### Simple phantom

**Figure 2:**
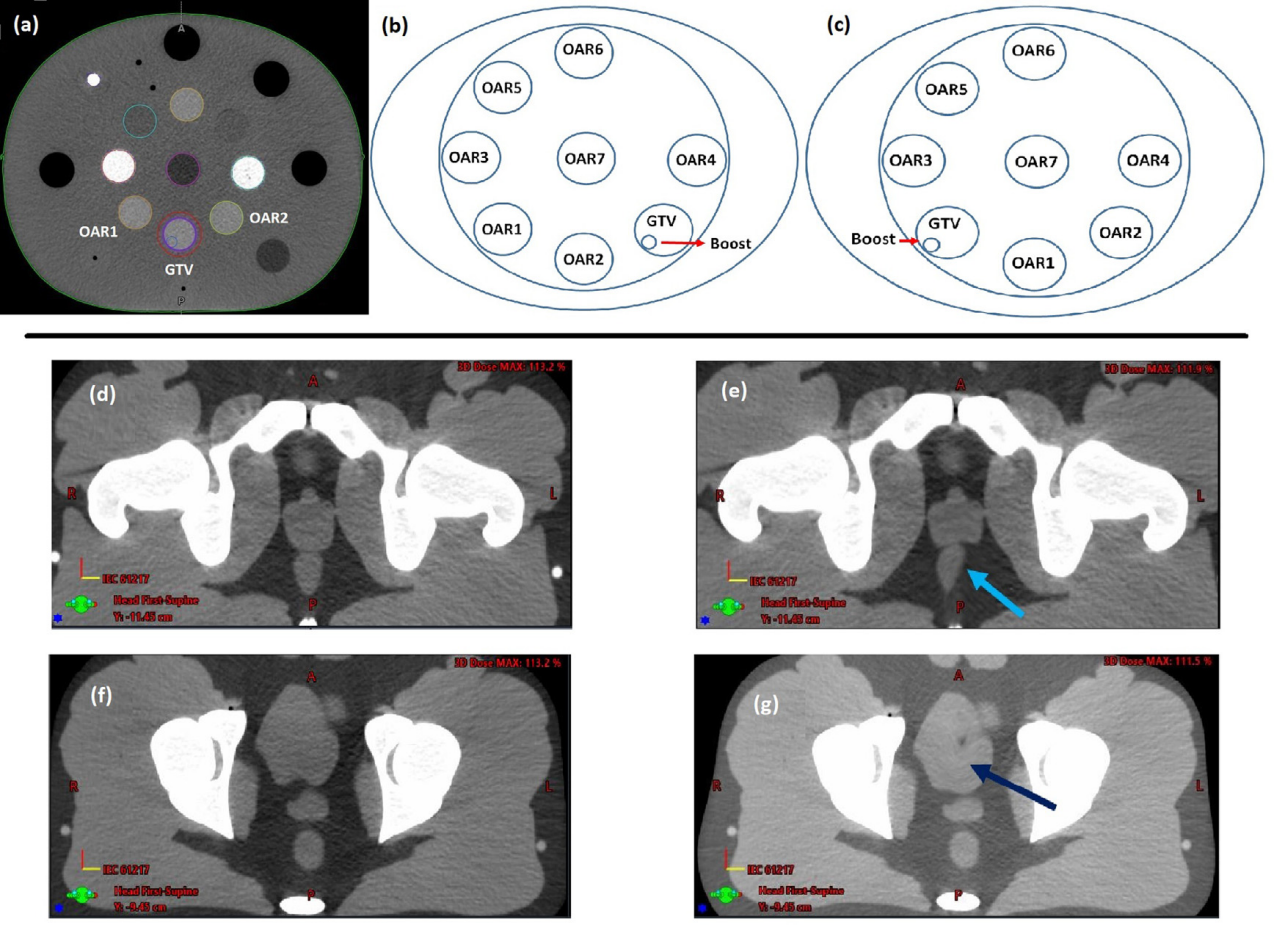
Contoured gross tumor volume, boost volume, and 7 organs at risk on electron density phantom for A, initial plan where target is at 6 o'clock and in 2 fraction adaptive treatments in which target was placed at B, 5 o'clock and C, 7 o'clock; D,F, original computed tomography scans and scans showing location that E, rectum and G, bladder are deformed.

An electron density phantom (CIRS Model 062M) was used for simple algorithm testing because of the wide availability of such phantoms in most clinics for periodic imaging QA. The phantom includes inner and outer sections, with each section including cylindrical cutouts to place inserts of known electron density. A CT scan of the electron density phantom was initially acquired with individual inserts contoured and associated with specific structures in the treatment planning system (TPS). Inserts in the inner section were assigned to be a target (gross tumor volume [GTV], contour drawn on insert at 6 o'clock), a boost volume (subvolume drawn inside GTV), and 7 OARs (other inserts), as shown in Figure 2. A PTV was generated within the TPS as a 0.5 cm expansion of the GTV.

Then, an initial plan was generated based on this CT scan and associated structure contours using the planning objectives shown in Table 1. Two treatment sessions with adaptation were performed using CBCT scans of the phantom acquired on the Ethos system. To trigger plan reoptimization, the target was intentionally drawn at different insert locations on each CBCT. In the first fraction, the target was recontoured at 7 o'clock (instead of 6 o'clock) and in the second fraction at 5 o'clock (Fig. 2). OAR1 and OAR2 were drawn at 6 o'clock in the first and second fractions, respectively.

**Table 1:** Planning objectives used in generating adaptive plans on electron density and pelvis phantom

Electron density phantom		
Organ	Objective	Priority
Planning target volume	D_98%_ ≥ 100%	1
	D_max_ < 110%	1
Gross tumor volume	D_99%_ ≥ 100%	1
Boost volume	D_99%_ ≥ 100%	1
OAR1, OAR2, and OAR7	D_max_ < 50%	2
OAR3, OAR4, OAR5, and OAR6	D_max_ < 25%	2

**Pelvis phantom**		
**Organ**	**Constraint**	**Priority**
Planning target volume	D_98%_ ≥ 100%	1
	D_max_ ≤ 110%	1
Bladder	V_50%_ < 30%	2
	V_80%_ < 8%	2
Rectum	V_50%_ < 17%	2
	V_80%_ < 7%	2
Bowel	V_65%_ < 30 cc	2
Left femur	V_65%_ < 5%	2
Right femur	V_65%_ < 5%	2

*Abbreviations:* D_x%_ = minimum dose delivered to x% of the volume; D_max_ = maximum dose received by the volume; OAR = organ at risk; V_x%_ = percent volume receiving at least x% of the prescribed dose.

Accumulated dose was updated by the system after each delivered fraction. To coarsely evaluate the accuracy of the accumulated dose, dosimetric parameters (including the mean structure dose) were derived manually from each session dose, and then summed and compared with the accumulated dose. Measurements were repeated 10 times to evaluate the stability and consistency of the results and obtain a range of variation. The accumulated dose was then manually calculated, and compared with the Ethos-generated dose to confirm that rigid transformations and DVFs were applied as intended by the treatment system.

The session dose and registration matrices, including a rigid transformation and DVF, were exported from the Ethos platform after delivery of each fraction. The transformed session/reconstruction dose was obtained first by applying the rigid transformation matrix. Then, the propagated dose was calculated by applying the DVF to the transformed reconstruction dose. The propagated doses were summed to generate the accumulated dose, and this manually derived accumulated dose was compared against the results from Ethos. Dose difference and gamma analysis with 2%/2 mm criteria were evaluated to compare the 2 accumulated doses.

As noted in Table 1, the target and OAR objectives have been assigned priority 1 (highest) and priority 2 (lower), respectively, in this study. In the Ethos TPS, clinical goals have priorities from 1 to 4, with 1 being the highest and 4 the lowest. Having target objectives with a higher priority than OARs has 2 implications. First, target coverage will be prioritized over OAR constraints. Second, the target is also prioritized over OARs in structure-guided deformation; thus, structures with a lower priority may not be deformed as accurately as the target. As a result, dose accumulation may be more accurate for the target compared to the OARs.

### Anthropomorphic phantom

The second set of measurements was performed for QA of the dose accumulation in a more realistic scenario using an anthropomorphic pelvis phantom (CIRS Model 801-P). A CT scan of the pelvic phantom was acquired first, and then simple deformations were applied to the CT scan to simulate organ changes for the sake of triggering adaptation on Ethos (Fig. 2).^29^ The deformations were generated by applying a custom vector field, created using the plastimatch toolbox.^26^ To simulate realistic deformation, we created a Gaussian vector field with the center of the Gaussian distribution located inside of either the bladder or rectum (OARs) adjacent to the prostate (target).

Then, the prostate, rectum, bladder, and bowels were contoured based on this deformed CT data set, and an initial plan was generated with plan objectives (Table 1). Two single-fraction adaptive plans were created and delivered. One additional single-fraction adaptive plan was also generated where no deformation was applied and the original planning CT was used. This additional plan was done as a control experiment in which there was no difference between the initial and adaptive plans. During the session, CBCT scans were acquired with the original phantom on the Ethos system. The prostate, small bowel, rectum, and bladder were contoured. Because of the changes in the anatomy relative to the synthetic pCT, plan adaptation was necessary to restore coverage. Then, the adapted plan was delivered on the phantom. Manual dose accumulation was performed after delivery with the same procedure as explained herein. Dose difference and gamma analyses were computed to verify the Ethos dose accumulation results. The dose difference and gamma analyses were evaluated after applying both rigid and deformable registrations to highlight the significance of deformation for this test case in the Ethos-specific adaptive workflow that produces sCT images.

## Results

### Simple phantom testing

**Figure 3:**
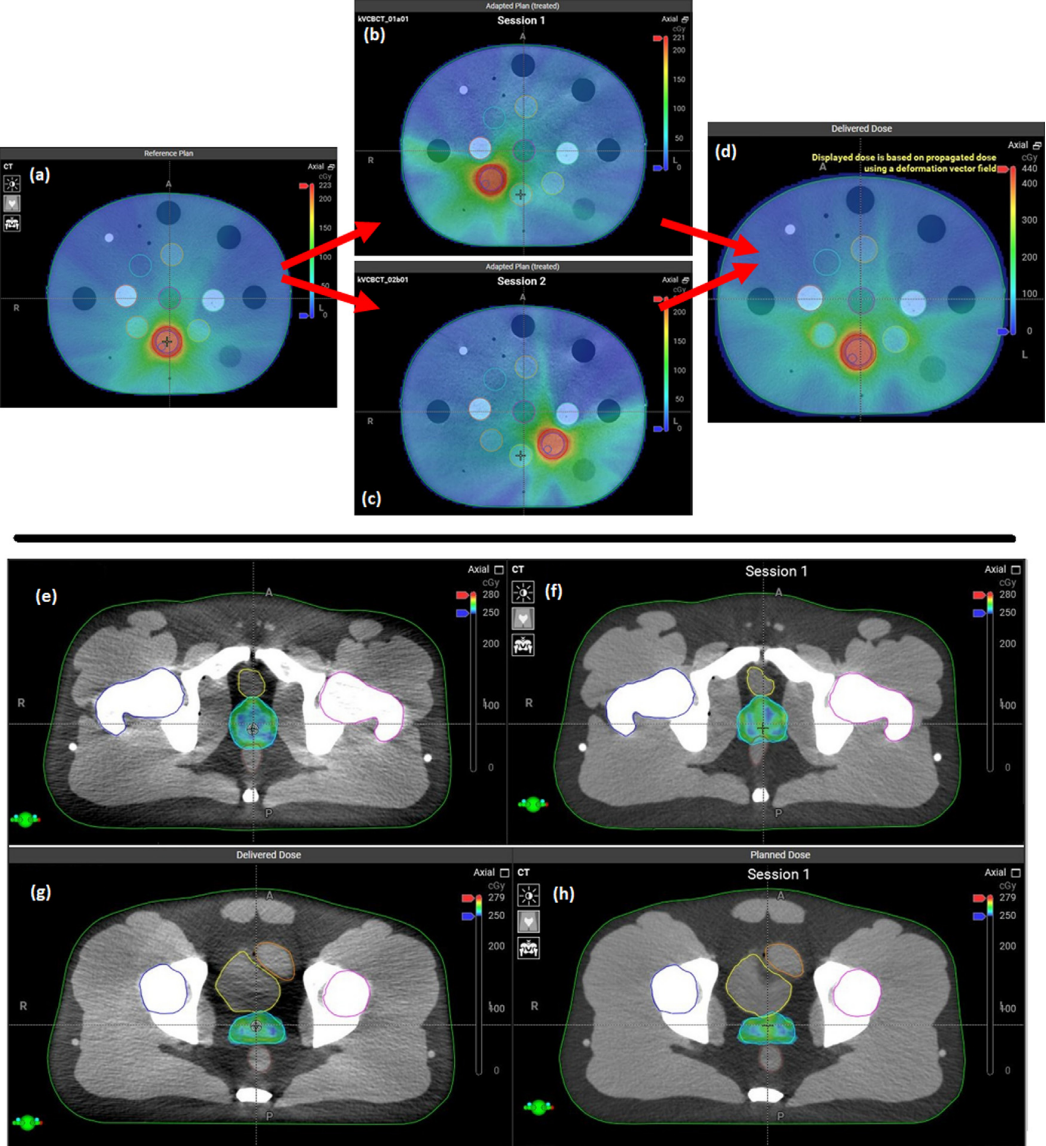
Dose distributions of A, initial plan; B,C, 2 adapted plans; and D, accumulated dose in electron density phantom. The dose distributions of the E,G, delivered adapted plans and F,H, initial plan of deformed E,F, rectum and G,H, bladder cases generated by Ethos system.

Figure 3 shows the dose distributions of the initial plan, 2 adapted plans, and accumulated dose generated by the Ethos system for tests performed with the electron density phantom. Table 2 compares the mean structure dose of the target and OARs as obtained by summing the mean dose between sessions and the system-generated accumulated dose in the electron density phantom for 10 repeated measurements.

**Table 2:** Median and range (median [minimum-maximum]) of percent difference in mean dose in gross tumor volume, boost volume, and 7 OARs in adaptive treatment (in which target was swapped) delivered on electron density phantom

	Fraction 1 dose, cGy (target at 5 o'clock)	Fraction 2 dose, cGy (targets at 7 o'clock)	Manual sum of delivered dose, cGy	Accumulated dose from Ethos, cGy	Difference, %
Gross tumor volume	218 (212-222)	217 (212-223)	435 (427-441)	435 (428-443)	0.0 (–0.5 to 1.8)
Boost	221 (217-224)	219 (216-225)	440 (434-446)	435 (433-446)	–0.2 (–1.8 to 0.0)
OAR 1	44 (38-58.2)	56 (45-60.6)	100 (83-118.8)	187 (177-218)	92.0 (62.0-113.3)
OAR 2	60 (40-70.6)	41 (33-50)	105 (89-113)	193 (150-198)	78.4 (58.3-114.4)
OAR 3	33 (24-37.2)	23 (20-30.2)	55 (44-67.4)	56 (45-68.1)	1.8 (1.0-2.3)
OAR 4	24 (21-32)	28 (24-33)	52.4 (48-57)	53 (49-56)	1.9 (–1.8 to 2.1)
OAR 5	29 (27-35)	25 (20-30)	53 (49-62)	54 (49-63)	0.0 (0.0-2.0)
OAR 6	31 (20-35)	33 (26-36.6)	63 (52-71.5)	63 (52-71.2)	0.0 (–1.5 to 1.6)
OAR 7	62 (57-71)	70 (49.1-73)	133 (117.1-138)	136 (120.2-142)	2.3 (1.5-2.9)

*Abbreviations:* OAR = organ at risk.

The vector fields derived from Ethos for both fractions are shown in Figure E1A. The results of gamma passing rates (GPRs) for the 3-dimensional dose comparison between the manually generated and Ethos-provided dose accumulations in the electron density phantom measurements are shown in Table 3. The maps of GPRs are shown after applying the rigid and deformable components of the registration matrices.

**Table 3:** Values and maps of GPRs for 3-dimensional dose comparison between manually generated and Ethos-provided dose accumulations in electron density and pelvis phantom

	GPR after applying rigid registration	GPR after applying deformable registration
**Electron density phantom**		

Target at 7 o'clock	59.98%	99.24%
Target at 5 o'clock	64.33%	99.59%
Total accumulation	78.32%	99.65%
Target at 7 o'clock		
**Pelvis phantom**		

No change	79.87%	99.1%
Rectum deformed	79.96%	99.32%
Bladder deformed	78.88%	99.30%
Rectum deformed		

*Abbreviations:* GPR = gamma passing rate.

### Pelvis phantom testing

Figure 3 shows the dose distributions of the delivered adapted plans and the initial plan of the deformed rectum and bladder cases generated by the Ethos system. The results of the GPRs for the 3-dimensional dose comparison between the manually generated and Ethos-provided dose accumulations in the pelvis phantom measurements are shown in Table 3. The 3-dimensional dose comparison in the form of GPR maps between the 2 accumulated doses for the case where the rectum was deformed are also shown in Table 3. The maps of GPRs are shown after applying the rigid and deformation components of the registration matrices.

## Discussion

As mentioned, dose accumulation is important to assess adaptive treatment; however, only limited commercial platforms provide this capability. The Ethos platform is one of few systems that support this feature but requires a QA program. Phantom approaches provide a direct and easy way to implement QA solutions. The goal of this study was to develop a routine QA method for the dose accumulation technique provided by Ethos during online treatment adaptation. Two phantoms were selected to perform the QA, including an electron density phantom and an anthropomorphic pelvis phantom. Table 3 shows that agreement is >2% for the mean dose in the GTV and boost volume between manually summed and Ethos-generated dose accumulations for the 2 adaptive plans created for the electron density phantom. Similar results were obtained for the OARs (<3%) except for OAR1 and OAR2 (up to 115%) whose positions were swapped with the target in the adaptive plans.

These results are consistent with vendor specifications given that in our plan design, the target had a higher priority over OARs in structure-guided deformation, and thus, higher dose accumulation accuracy. A contributing factor to the apparently poor results for OAR1 and OAR2 could include the unrealistic, discontinuous change in location between these structures and the target. These tests based on a common commercial phantom highlight limitations in the investigated dose accumulation platform. Such repeated measurements could be performed at the time of commissioning, and used to set an accuracy baseline for targets and OAR3 to OAR7 for subsequent measurements that might be performed after a major upgrade or as part of periodic QA.

Table 3 show agreement between manually obtained and system-produced dose accumulation through a gamma analysis that indicates >99% GPR with 2%/2 mm criteria. The GPRs in Table 3 show that only applying the rigid component of the registration yields poor agreement (<80%), which highlights that deformation is significant in the dose accumulation for this test case due to factors including the creation of sCT and modeling of nonrigid anatomic change. It is interesting to note that the deformable component of the registration caused an approximate 20% increase on the GPR in the pelvis phantom, even on the controlled plan where no deformation had been applied and the pCT was similar to the CBCT. Further investigation is needed to verify the deformable registration between the CBCT and pCT.

## Conclusions

This study demonstrated agreement between manually obtained and TPS-generated accumulated doses in terms of both mean structure doses and local 3-dimensional dose distribution in 2 commonly used phantoms. Large disagreements were observed between the manually summed mean doses and the dose accumulations of OAR1 and OAR2. However, these discrepancies were expected based on the lower assigned priority to dose objectives relative to the target in addition to the unrealistic changes in position for these structures, specifically fraction by fraction. The straightforward tests described in this study can support periodic QA of dose accumulation tools for modern RT platforms that provide online treatment adaptation. Based on the given priorities to the target (priority 1) and OARs (priority 2) in the adapted plans, we suggest ±2% and ±3% thresholds as acceptable ranges of variation in the differences in mean dose of the target and OARs (other than the two that were swapped with the target) between the manually summed dose and Ethos-generated dose accumulation.

These tests confirm functionality of onboard dose accumulation algorithms, and we recommend these tests be performed at the time of acceptance and commissioning to establish a baseline according to vendor specifications, as well as after any major system upgrades to test the functionality and consistency of the dose accumulation tool. Given their simplicity, these tests could be added also to a periodic QA program at low frequency to evaluate the consistency of the results. In vivo dose accumulation cannot directly be verified, but our methods can ensure that tools supporting dose accumulation, such as deformable image registration, are functioning consistently to provide estimates of accumulated doses. In our future work, we plan to implement these tests in our QA program, and monitor the results over time to evaluate stability and consistency and assess any meaningful variation in clinical implementation of dose accumulation.

## Appendix Supplementary materials

Supplementary materials Supplementary material associated with this article can be found, in the online version, at [comp will supply]. Image, image 1
