# Supplementary figures and images for: Phantom-based Quality Assurance of a Clinical Dose Accumulation Technique Used in an Online Adaptive Radiation Therapy Platform
Source: Adv Radiat Oncol. 2022 Dec 6;8(3):101138. doi: 10.1016/j.adro.2022.101138 (PMC9860416; doi:10.1016/j.adro.2022.101138)

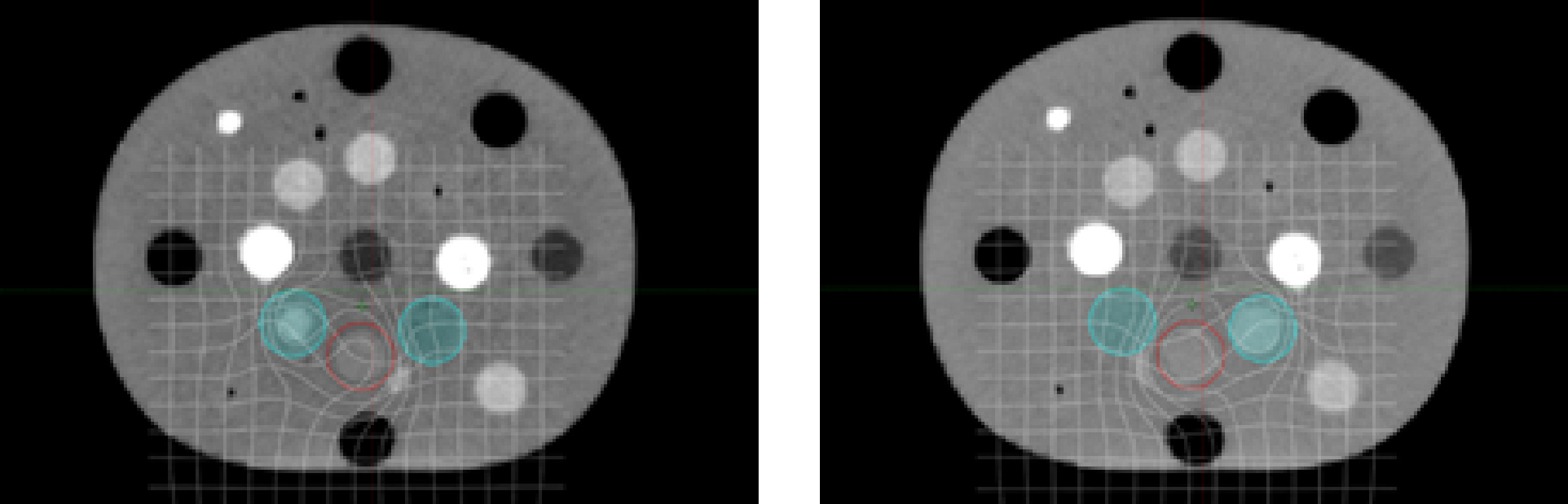

Supplement: Supplementary file 1 — Supplementary materials Supplementary material associated with this article can be found, in the online version, at [comp will supply]. [file mmc1.jpg]
